# Disease-associated CAG·CTG triplet repeats expand rapidly in non-dividing mouse cells, but cell cycle arrest is insufficient to drive expansion

**DOI:** 10.1093/nar/gku285

**Published:** 2014-04-21

**Authors:** Mário Gomes-Pereira, James D. Hilley, Fernando Morales, Berit Adam, Helen E. James, Darren G. Monckton

**Affiliations:** 1 Institute of Molecular, Cell and Systems Biology, College of Medical, Veterinary and Life Sciences, University of Glasgow, Glasgow G12 8QQ, UK; 2 Inserm UMR 1163, Laboratory of *CTG Repeat Instability and Myotonic Dystrophy Type 1*, 75015 Paris, France; 3 Paris Descartes-Sorbonne Paris Cité University, Imagine Institute, 75015 Paris, France; 4 Instituto de Investigaciones en Salud y Escuela de Medicina, Universidad de Costa Rica, San José, Costa Rica

## Abstract

Genetically unstable expanded CAG·CTG trinucleotide repeats are causal in a number of human disorders, including Huntington disease and myotonic dystrophy type 1. It is still widely assumed that DNA polymerase slippage during replication plays an important role in the accumulation of expansions. Nevertheless, somatic mosaicism correlates poorly with the proliferative capacity of the tissue and rates of cell turnover, suggesting that expansions can occur in the absence of replication. We monitored CAG·CTG repeat instability in transgenic mouse cells arrested by chemical or genetic manipulation of the cell cycle and generated unequivocal evidence for the continuous accumulation of repeat expansions in non-dividing cells. Importantly, the rates of expansion in non-dividing cells were at least as high as those of proliferating cells. These data are consistent with a major role for cell division-independent expansion in generating somatic mosaicism *in vivo.* Although expansions can accrue in non-dividing cells, we also show that cell cycle arrest is not sufficient to drive instability, implicating other factors as the key regulators of tissue-specific instability. Our data reveal that *de novo* expansion events are not limited to S-phase and further support a cell division-independent mutational pathway.

## INTRODUCTION

At least 17 inherited human neurological disorders are caused by the expansion of genetically unstable DNA trinucleotide repeats (1,2). Most of these disorders involve a CAG·CTG repeat expansion, such as Huntington disease (HD) and myotonic dystrophy type 1 (DM1). Longer inherited CAG·CTG repeat alleles cause more severe symptoms and an earlier age of onset (2). Expanded alleles are highly unstable in the germline and show a marked bias toward additional gains in repeat number, thus accounting for the decreasing age of onset and increasing disease severity in successive generations (anticipation). Expanded CAG·CTG repeats are also somatically unstable in a process that is age-dependent, tissue-specific and expansion-biased, and mediated by multiple small gains and losses in repeat number (3,4). In particular, very large expansions accumulate in the muscle of DM1 patients (5) and in the striatum of HD patients (6), the two major affected tissues in these disorders. Moreover, higher individual-specific repeat expansion rates have been directly linked with increased disease severity and earlier age of onset in HD and DM1 (7,^8^). These data strongly implicate somatic expansion in the tissue-specificity and progressive nature of the symptoms (2).

Multiple pathways of DNA metabolism have been implicated in generating repeat expansions in mammalian cells, such as replication (9–11), mismatch repair (12–16), base excision repair (17), nucleotide excision repair (18) and transcription (19,20). Most clear is the requirement of functional mismatch repair (MMR) proteins for the accumulation of somatic expansions (12–16). Although it has been proposed that inappropriate MMR of alternative DNA structures might operate independently of cell division (14), MMR is more intimately linked with DNA replication and it has been suggested that MMR proteins may act instead to stabilize slipped strand DNA intermediates arising during replication (21,22). Replication slippage has long been assumed to be an important mechanism for generating expansions (23) and a primary role for DNA replication and cell division through DNA polymerase slippage is supported by data generated in bacteria and yeast model systems (21,24–25). The replication slippage model predicts that cell division is required to generate expansions and that expansions will accrue at a faster rate in tissues with a high cell turnover. These predictions are at odds with data derived from HD and DM1 patients (6,26) and from numerous transgenic mouse models (27–30) in which there is no obvious correlation between the somatic expansion rate of the DNA and the proliferative capacity of the tissue. However, such correlative studies are limited by the complex nature of tissues, which are comprised of multiple cell types with differing proliferative capacities, and our inability to define the replicative history of any given cell *in vivo.* In fact, the expansion rates of unstable trinucleotide repeats carried by the same cell type have not been directly compared between proliferating and non-proliferating cultures. As a result, despite some circumstantial data, no definitive evidence exists for the continuous accumulation of expansions over time in homogeneous populations of non-proliferative cells. Indeed, it has been suggested that DNA replication during genome duplication and cell division is necessary to initiate expansion in DM1 patient fibroblasts (11).

To explore the role of the cell cycle in mediating expansions, we previously generated a cell culture model that reproduces time-dependent, expansion-biased tissue-specific somatic mosaicism (31) derived from a transgenic mouse model of unstable CAG·CTG repeats (28). Interestingly, the cell type-specific expansion rates measured in different cultures could not be accounted for by differences in cell division rates (32). For instance, the repeat tract continued to expand rapidly in a kidney cell line (D2763K), but was faithfully replicated without mutation for over 100 population doublings (PDs) in a lung cell line (D2763L). These data demonstrated that cell division was insufficient to drive expansion, but did not rule out the possibility that DNA replication and cell cycle progression were nonetheless required to mediate expansions. Here we sought to test whether cell division is absolutely necessary for trinucleotide repeat instability in somatic cells through the establishment of a cell culture model of replication-independent repeat instability. The direct comparison of trinucleotide repeat expansion rates in the same cell type growing under proliferating and non-proliferating conditions would address this question.

## MATERIALS AND METHODS

### Mouse cell culture and chemical cell cycle arrest

All experiments were performed in accordance with the UK Home Office Animals Scientific Procedures Act 1986 and with the guidelines of the host institution for the welfare of animals. *Dmt-*D transgenic mice carry an expanded CAG·CTG repeat surrounded by ∼750 bp of flanking DNA from the human *DM1* locus (33,34). D2763Kc2 (31) and D2763L (32) cells were maintained and passaged as previously described (31,32). For chemical cell cycle arrest experiments, a progenitor culture was split into multiple aliquots: six no-drug replicate controls and for each cell-cycle inhibitor, six replicate treated cultures (Supplementary Figure S1). All cultures were maintained in parallel throughout the course of the experiment (up to 121 days) and supplied with fresh medium every two to three days. Treated cultures were continuously exposed to the chemicals, with the exception of mitomycin C (MMC) arrested cells, which were exposed to 30 μM MMC for 180 minutes, and subsequently maintained in standard medium. Dividing control cells were passaged when confluent, at a 1:40 dilution. Cell viability was periodically determined by trypan blue exclusion assays following phosphate buffered saline (PBS) washing, which eliminated the majority of semi-detached dead cells. Single-cell clones were obtained by limiting dilution of progenitor cultures. Cell cycle progression was determined by bromodeoxyuridine (BrdU) incorporation assays and proliferating cell nuclear antigen (PCNA) immunofluorescence (see Supplementary Methods).

### p16^INK4a^ and p21^WAF1^ overexpression and cell cycle arrest

Two progenitor cultures expressing either inducible p16^INK4a^ or p21^WAF1^ were split into 12 parallel cultures. Six proliferating controls were maintained in standard growth medium in parallel with six cultures continuously exposed to 10 nM mifepristone A to induce p16^INK4a^ or p21^WAF1^ expression. Treated and control cells were maintained as for the chemical treatment experiments for 89 days following mifepristone A induction. To control for any effect of pSwitch expression on repeat metabolism, a pSwitch-transfected progenitor culture was divided into 12 parallel cultures: six no-mifepristone A controls and six ‘induced’ controls continuously exposed to 10 nM mifepristone A.

### MEF cell culture and cell cycle arrest by serum starvation

Progenitor *Dmt*-D mouse embryonic fibroblast (MEF) cultures were prepared as previously described (14) and split into 12 aliquots and cultured to confluence. Six replicate cultures were arrested by serum starvation in 0.1% (v/v) fetal bovine serum and six dividing control cells were maintained in 10% (v/v) fetal bovine serum and passaged when confluent, at a 1:4 dilution, for up to 139 days.

### Protein sample preparation and western blotting

Protein samples were extracted and analyzed as previously described (14). See Supplementary Methods for detailed western blot protocols and antibody dilutions.

### Small pool PCR amplification

Cell cultures were washed twice with PBS to remove semi-detached dying/dead cells. Cultured cells were collected by trypsin digestion and DNA samples were extracted using a Nucleon DNA extraction kit for blood and tissue culture (Nucleon) following the manufacturer's protocol (32). Repeat length variability in each sample was assessed by small pool PCR (SP-PCR) analysis using oligonucleotide primers DM-C and DM-BR, as previously described (3,31). The analysis of somatic mosaicism by SP-PCR and the representation of repeat number variation is described in the Supplementary Methods.

### Statistical analyses

Statistical analyses were performed using Microsoft Excel and MINITAB for Windows (release 14.1, 2003, Minitab Inc.).

## RESULTS

### Chemical arrest of the cell cycle

**Figure 1.**
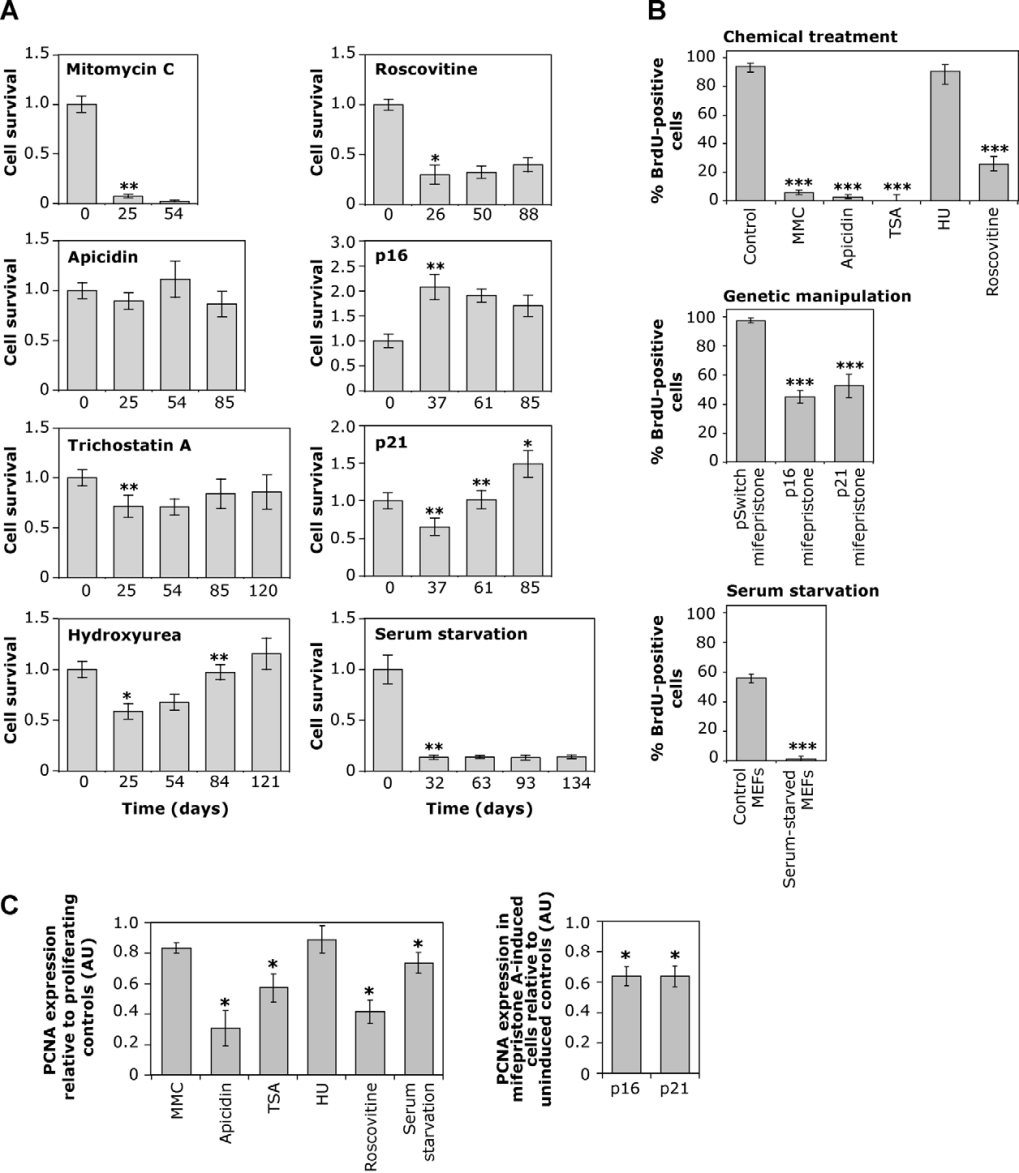
Analysis of cell cycle arrest. (**A**) Cell culture dynamics. The graphs show mean cell survival (±SD) of six replicate cultures over time. Cell survival of chemically treated cultures, cells expressing p16^INK4a^ and p21^WAF1^ following mifepristone A induction, and serum-starved MEFs, was calculated relative to the day zero culture. Statistically significant differences between two consecutive time points are illustrated with asterisks (*, *P* < 0.05; **, *P* < 0.01; Mann–Whitney *U* test). (**B**) Proportion of BrdU positive cells. The histograms show the percentage of BrdU immunopositive cells and the error bars indicate the 95% confidence intervals for control and treated cultures. Statistically significant reductions in BrdU staining relative to controls are indicated (***, *P* < 0.001; Fisher's exact test). (**C**) Relative PCNA expression levels. The graph on the left shows the quantitative analysis of PCNA protein expression levels in arrested cells relative to the corresponding proliferating controls. The graph on the right shows the quantitative analysis of PCNA protein expression (±SD) in mifepristone A-induced cells, co-transfected with pSwitch regulatory plasmid and pGENE/V5-His A/p16^INK4a^ (p16) or pGENE/V5-His A/p21^WAF1^ (p21), relative to un-induced transfected controls. Statistically significant reductions in PCNA protein levels relative to proliferating controls are indicated (*, *P* < 0.05; Mann–Whitney *U* test). AU, arbitrary units.

**Figure 2.**
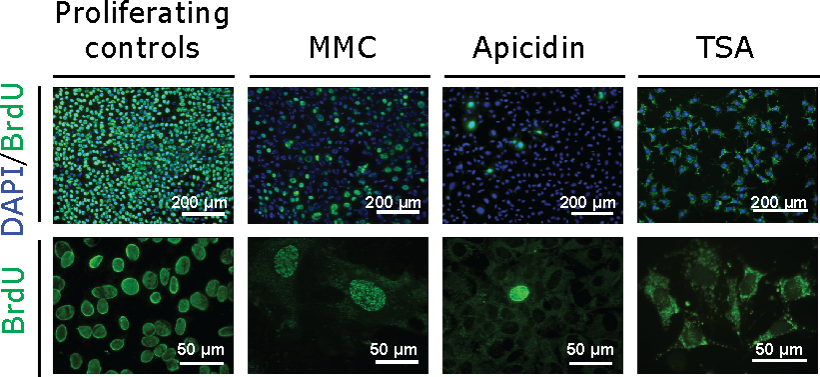
BrdU incorporation patterns. To measure levels of DNA synthesis we performed a BrdU incorporation assay. Representative low magnification images (left) reveal the relative proportion of BrdU immunostaining (green) nuclei counter-stained with DAPI (blue). Representative high-magnification images (right) illustrate BrdU immunostaining patterns within individual cells and reveal differences between chemical treatments.

To develop a non-proliferative cell model of repeat size instability and to determine directly if DNA replication and cell division are required to generate trinucleotide repeat expansions, we used chemical inhibitors to arrest a clonal transgenic mouse cell line at various phases of the cell cycle. The D2763Kc2 cell line was selected to perform this study because it carries an unstable CAG·CTG repeat that expands rapidly with time, recreating the step-wise, expansion-biased somatic instability of trinucleotide repeat expansions (31). These cells provide a suitable model system to investigate the core mechanisms of trinucleotide repeat dynamics and identify factors that modify repeat expansion rates. Cells were arrested in S and G_2_/M by exposure to MMC (35), in S with hydroxyurea (HU) (36), in G_1_ with roscovitine (37), and in G_1_ and G_2_ with apicidin and trichostatin A (TSA) (38,39). For each chemical treatment, six replicate cultures of arrested cells were maintained for up to 121 days in parallel with six replicate control cell cultures (Supplementary Figure S1). To monitor the effect on cell cycle progression, we carefully examined the number of viable cells by trypan blue exclusion assays, the levels of DNA synthesis using BrdU incorporation and protein levels of PCNA (Table 1, Figure 1, Supplementary Figures S2 and S3). The rapidly proliferating control cells (population doubling time, PDT ∼25 h) expressed high levels of PCNA in the nucleus and nearly all cells incorporated high levels of BrdU (94%). The number of viable cells decreased significantly following the initial period of exposure to the chemical, as a result of high mortality, and it remained low throughout the treatment. No additional signs of cell death were detected (e.g. overt cell detachment, cell membrane breakdown, cell/organelle swelling) as the treatment progressed, hence having minimal impact on the PDT measured. Viable cells did not accumulate in any treated cultures and only in HU-treated cultures was there an increase in viable cell numbers at the end of the experimental period (Figure 1A). All the other chemical treatments resulted in highly statistically significant reductions in the rate of incorporation of BrdU (Figure 1B, Supplementary Figure S2) and dramatic decreases in PCNA protein levels (Figure 1C, Supplementary Figure S3). In particular, homogenous nuclear incorporation of BrdU was not detected in TSA-treated cells and was only observed at very low levels (<2%) in apicidin-treated cells, possibly as a result of a small fraction of cells that escaped cell cycle arrest. None of the MMC-treated cells displayed the homogenous nuclear incorporation of BrdU typical of the dividing cells, but a low proportion (∼6%) did present with discrete nuclear BrdU-positive intranuclear foci (Figure 2). Such foci have been observed previously in MMC-treated cells and shown to be sites of active DNA repair (40). The punctuated cytoplasmic staining in apicidin- and TSA-treated cells likely results from BrdU incorporation into mitochondrial DNA (41). Consistent with an S-phase arrest, HU-treated cells showed only a slight drop in PCNA expression and BrdU incorporation (91%) following exposure to the chemical for one week. However, HU-treated cultures displayed a dramatic reduction in cell number after 25 days that only slowly recovered with time and only exceeded starting cell numbers at the end of the treatment period (121 days) (Figure 1A). HU-treated cells thus continued to divide, but with a dramatically increased PDT of ∼120 days. Thus, all of the chemical treatments resulted in either complete cell cycle arrest, or at least a 100-fold increase in the PDT.

**Table 1.** Cell culture growth and expanded CAG·CTG repeat dynamics

Treatment [drug concentration]	Time [days]	PDs	PDT [h]	BrdU incorporation [%] (*P*)^a^	Median repeat gain relative to progenitor [repeats] (*P*)^b^	Median rate of expansion [repeats per day] (*P*)^c^
Control 1^d^	121	120	24.2	94	65 (<0.0001)	0.54
Mitomycin C [30 μM]^e^	54	<1	>3000	6 (<0.0001)	68 (<0.0001)	1.26 (0.005)
Apicidin [320 nM in 0.1% DMSO]	85	<1	>3000	2 (<0.0001)	39 (<0.003)	0.46 (0.066)
Trichostatin A [160 nM in 0.1% DMSO]	120	<1	>3000	0 (<0.0001)	68 (<0.0001)	0.56 (0.81)
Hydroxyurea [50 μM]	121	∼1	∼3000	91 (<0.21)	55 (<0.0001)	0.45 (0.02)
Control 2^d^	89	100	21.4	94	42 (<0.018)	0.48
DMSO [0.1%]	89	95	22.5	N/D	37 (<0.0004)	0.41 (0.47)
Roscovitine [20 μM in 0.1% DMSO]	89	<1	>3000	26 (<0.0001)	27 (<0.016)	0.33 (0.30)
pSwitch control	89	69	31.0	N/D	45 (<0.0007)	0.50
pSwitch mifepristone A [10 nM]	89	68	31.4	97	92 (<0.0001)	1.04 (0.005)
p16 control	89	74	28.9	N/D	46 (<0.032)	0.52 (0.94)
p16 mifepristone A [10 nM]	89	∼2	∼1000	45 (<0.0001)	83 (<0.0001)	0.93 (0.008)(0.39)^f^
p21 control	89	69	31.0	N/D	45 (<0.0007)	0.51 (0.69)
p21 mifepristone A [10 nM]	89	<2	>1000	52 (<0.0001)	69 (<0.0055)	0.77 (0.38)

^a^Each treatment sample was compared to the relevant control sample using a one-tailed Fisher's exact test.

^b^Repeat length distributions in each replicate were compared to the progenitor culture using a one-tailed Mann–Whitney *U* test. The maximum *P*-value obtained across all the replicates for each treatment is shown.

^c^Median repeat lengths for each treatment were compared to the relevant control culture using a two-tailed Mann–Whitney *U* test as previously described (31).

^d^Chemical cell cycle arrest of cells was performed in two independent experiments, one for MMC, apicidin, TSA and HU treatment, and one for roscovitine treatment.

^e^Single acute exposure.

^f^Compared relative to the pSwitch mifepristone A control.

### CAG·CTG repeats continue to expand in chemically arrested cells

**Figure 3.**
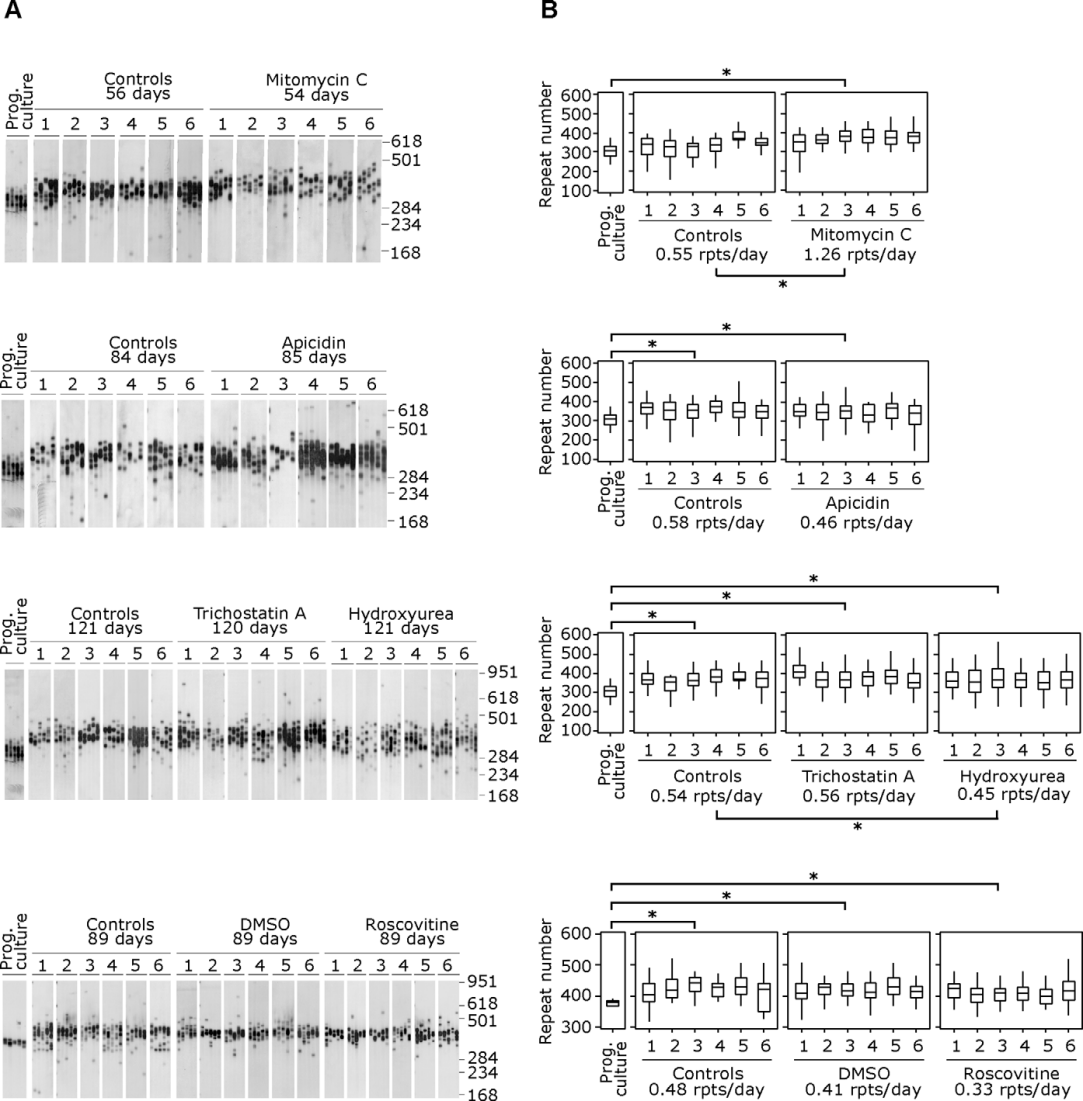
The expanded CAG·CTG repeat continues to expand in chemically arrested cells. (**A**) Representative SP-PCRs of the expanded CAG·CTG repeat in replicate D2763Kc2 cell cultures (1-6)

The effect of cell cycle arrest on the dynamics of the expanded CAG·CTG repeat was assessed by sensitive SP-PCR-based approaches (3) to measure repeat length variation in progenitor cultures, rapidly proliferating controls cells, solvent controls and chemically arrested cell cultures (Figure 3A, Supplementary Figure S4). The quantitative analysis of single molecule SP-PCR products allowed accurate assessment of average repeat sizes before, during and after chemical treatment. We calculated rates of repeat expansion by dividing the median increase in repeat size of each replicate culture by the time in culture (31). The results revealed that, as expected, the repeat expanded rapidly in the control proliferating cells, gaining an average of ∼0.5 repeats per day (Figure 3B). The repeat expansion rate was not altered by DMSO, the solvent used for histone deacetylase (HDAC) inhibitors (Table 1). Remarkably, the average repeat tract length in all six replicates increased relative to the progenitor culture, throughout the course of the experiment, in all five chemical approaches to cell cycle arrest (*P* < 0.02 in each replicate; Mann–Whitney *U* test; Figure 3, Supplementary Figure S4). These data demonstrate directly that expanded CAG·CTG repeat tracts can continue to expand in the absence of cell division. Notably, with four out of the five treatments, the rate of expansion per unit of time was at least as great as observed in the rapidly dividing control cells (*P* > 0.05; Mann–Whitney *U* test). Indeed, despite having the least effect on the levels of DNA synthesis and cell cycle progression, HU was the only treatment to result in a detectable decrease in the expansion rate relative to the dividing controls (rates were reduced by ∼15%; *P* = 0.02; Mann–Whitney *U* test). Interestingly, the rate of expansion in MMC-arrested cells was dramatically increased (>1.2 repeats per day; *P* = 0.005; Mann–Whitney *U* test).

### CAG·CTG repeat size gains in genetically arrested cells

**Figure 4.**
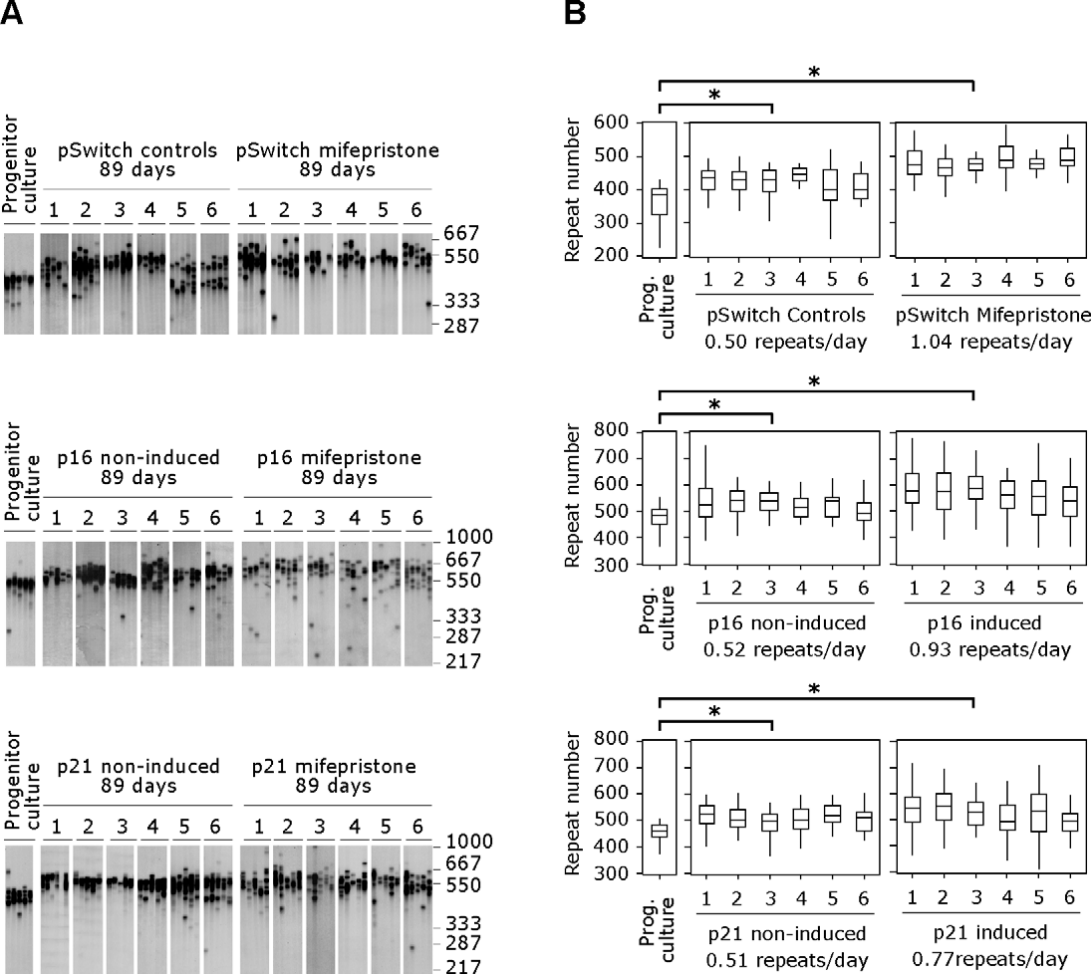
The CAG·CTG repeat expansion continues to expand in genetically arrested cells. (**A**) Representative SP-PCRs of the expanded CAG·CTG repeat in replicate D2763Kc2 cell cultures (1-6)

Although the variety of different cellular pathways targeted by the chemical treatment used argues against it, we considered it formally possible that the cell division-independent expansions observed were an artifact of chemical treatments. We therefore sought to establish if the repeat continued to expand in cells arrested using more physiologically relevant approaches. D2763Kc2 cells were arrested for up to 89 days by overexpressing the cyclin-dependent kinase inhibitors p16^INK4a^ or p21^WAF1^ (42), using a mifepristone-inducible gene expression system in six replicate cultures maintained in parallel with six un-induced controls (Supplementary Figures S1 and S5). BrdU incorporation levels (*P* < 0.0001; Fisher's exact test) and PCNA expression (*P* < 0.05; Mann–Whitney *U* test) were both greatly reduced, and cell numbers either increased very slowly with time (p21 expression) or initially increased slightly and then decreased with time (p16 expression) (Figure 1A). Although neither cell line was completely arrested throughout the course of the experiment, PDTs were increased by ∼40-fold relative to un-induced controls (Table 1). As with the chemical approach to cell cycle arrest, the repeat continued to expand (*P* < 0.006; Mann–Whitney *U* test) in all six replicates from both p16 and p21 genetically arrested cells at rates at least as great as those of control cells (*P* >> 0.05; Mann–Whitney *U* test) (Table 1, Figure 4). Indeed, the expansion rates of the induced p16-expressing cells were significantly higher than p16 un-induced controls (*P* = 0.008), but were no greater than in mifepristone-treated, ‘induced’ pSwitch controls (Table 1).

To further discard the possibility of genotoxic effects of chemical exposure as the major source of repeat expansion in arrested D2763Kc2 cells, we monitored repeat dynamics in a small number of primary kidney cultures arrested by mild contact inhibition or serum starvation (Supplementary Figure S6). The analysis revealed the continuous accumulation of larger repeat alleles in non-dividing cells in the absence of chemical treatment or protein overexpression. In summary, ongoing repeat size gains in cells arrested by overexpression of negative regulators of cell cycle progression or by mild contact inhibition and serum starvation provides additional support for the dissociation between repeat expansion and cell division.

### Cell cycle arrest does not initiate triplet repeat instability

**Figure 5.**
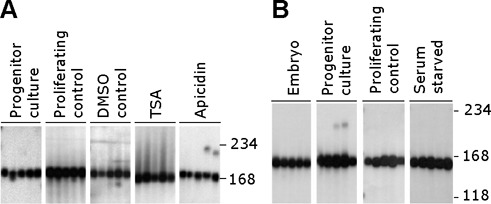
Cell cycle arrest is not sufficient to mediate CAG·CTG repeat expansion. Representative SP-PCRs of the expanded CAG·CTG repeat in chemically arrested D2763L lung cell cultures (**A**) and MEF cell cultures arrested by serum starvation (**B**) are shown. Also included are the relevant dividing cell controls, the embryo from which the MEF line was established, the progenitor culture from which all cultures were derived at day zero and the vehicle DMSO control. For clarity, only one representative replicate from each set of six replicate cultures, and data from only one of the three MEF cell lines analyzed, is shown. D2763L lung cells were treated for 90 days with 500 nM apicidin or 100 nM trichostatin A, or for 45 days with 30 μM MMC. The scale on the right indicates the DNA molecular weight markers converted into number of CAG·CTG repeats.

Using both chemical and genetic induction of cell cycle arrest, we have shown that an expanded CAG·CTG repeat continues to expand in cells arrested at various stages of the cell cycle including S, G_1_, G_2_ and G_2_/M at rates that are at least equivalent to those observed in dividing cells. These data indicate that expansions are not limited to S-phase, excluding DNA polymerase slippage during replication as the primary initiating event. Interestingly, the repeat expansion rate was significantly higher under some conditions of cell cycle arrest. Combined with the apparent preferential accumulation of CAG·CTG expansions in tissues enriched for post-mitotic cells *in vivo* (6,26,30), these data raise the intriguing possibility that cell cycle arrest may be an initiating event in mediating expansions. We tested this hypothesis by using TSA, apicidin and MMC to arrest D2763L lung cells in which the repeat is normally stably maintained (32). Cell cycle arrest failed to stimulate repeat expansion in these cells (Figure 5A). These results also demonstrate that the continuous accumulation of repeat size variability in D2763Kc2 cells arrested by chemical exposure cannot be accounted for by a major genotoxic effect of the drugs used on DNA metabolism. Likewise, serum starvation induced cell cycle arrest (Figure 1), but did not destabilize the expanded CAG·CTG repeat tract in three independent *Dmt*-D transgenic MEF cell lines (Figure 5B). These data indicate that neither DNA replication nor cell cycle arrest, *per se*, are necessary or sufficient to initiate expansion and establish that the major initiating event for repeat expansion in these cells is cell cycle-independent. Given the identical *cis* context of the expanded CAG·CTG repeat tract in all the cell lines analyzed, these results predict a major role for *trans*-acting tissue-specific factors independent of cell cycle dynamics.

## DISCUSSION

Here, by monitoring repeat size variability over time in arrested cultures we have demonstrated directly that trinucleotide repeats continue to expand in non-dividing mammalian cells. This effect was consistently observed in cells arrested at different stages of the cell cycle through a variety of chemical and genetic approaches. These data unequivocally establish that expansions can occur independently of cell cycle progression and are not limited to S-phase and are thus not linked to genome duplication. Strikingly, the rates of expansion observed were similarly high in both dividing and non-dividing cells. Indeed, only exposure with HU reduced the expansion rate relative to dividing cells, and this was the treatment that had the least effect on progression through the cell cycle, possibly inducing S phase stasis (43). The minor BrdU incorporation following other treatments argues against S phase stasis and significant genomic DNA duplication in treated cells. MMC, which precipitated a robust cell cycle arrest, actually resulted in a more than 2-fold increase in the expansion rate. In all the other treatments, the rates of expansion were comparable in dividing and non-dividing cells. Although these data do not preclude a role for cell division-dependent replication slippage in mediating expansions in the dividing cells, the simplest explanation is that cell division *per se* has little effect on the rate of repeat expansion and that the repeat size gains occur in a time-dependent manner in both the dividing and non-dividing cells. *In vivo* support for a cell division-independent expansion mechanism is afforded by several lines of circumstantial evidence, not least of which is the observation of large expansions in somatic tissues enriched for post-mitotic cells such as muscle in DM1 patients (26) and brain of HD patients (6), and in a variety of ostensibly post-mitotic tissues of transgenic mouse models of unstable CAG·CTG repeats (27–28,30). More directly, significant repeat gains were observed in apparently terminally differentiated neurons physically selected from human and mouse HD brains (44,45). However, the activation of neurogenesis in neurodegenerative disorders (46) confounds the dissociation between cell division and repeat expansion in HD neurons.

Very interestingly, the integration of data on tissue-specific somatic mosaicism in HD transgenic mice with high throughput gene expression data revealed a negative correlation between cell cycle pathways and the degree of repeat instability of a tissue (47), consistent with a cell cycle-independent expansion mechanism. Indeed, these authors proposed that cell cycle arrest might even promote instability and provided some data in support of this idea (47). To further test this hypothesis we arrested both an established mouse lung cell line and primary MEFs, in which the exact same CAG·CTG-containing transgene is stably maintained during cellular proliferation. The expanded triplet repeat was not destabilized in these arrested cells, suggesting that exit from the cell cycle alone is not sufficient to initiate instability. Our data are consistent with *in vivo* repeat stability in regions of the brain, such as the cerebellum, in which only very low levels of mosaicism are usually observed even though many of the cells are quiescent (6). These data do not preclude a role for exit from the cell cycle in modifying repeat dynamics, but do confirm that the cell cycle is not the only factor in driving instability and that other *trans*-acting factors must also be important in defining the tissue specificity of expansion.

Most of the support for a replication-dependent mechanism of trinucleotide repeat instability comes from microorganisms (21,24–25) and mammalian cell models (10,48,49) in which the bias toward expansions observed in humans is not reproduced. The relevance to the repeat dynamics in humans of observations made in such models remains unknown. The apparent stabilizing effect of cell cycle arrest by serum starvation on the expanded repeat in DM1 fibroblasts (11) likely reflects the low rate of expansion observed in these cells and the insensitive detection methods used. Similarly, the apparent destabilizing effect of polymerase inhibitors on human cell lines carrying CAG·CTG repeat expansions has been interpreted to provide support for a replication-dependent mutation pathway (10,11). However, the low doses of polymerase inhibitors used were insufficient to block cell cycle progression, but did lengthen PDTs. It is thus possible that the increase in the rate of expansion per PD was mediated by an indirect effect on cell cycle dynamics, and that the rate of expansion per unit of time was not affected.

While DNA duplication is limited to S-phase, DNA repair occurs at all stages of the cell cycle and is thus a good candidate to facilitate repeat expansion. Indeed, the requirement of DNA MMR genes, such as *Msh2* (13,16), *Msh3* (12,50) and *Pms2* (14) to mediate repeat expansions, combined with the data presented here, supports an alternative mutation mechanism to replication slippage that is based on inappropriate DNA MMR (14). In this model, MMR proteins process alternative DNA structures formed within repeat tracts to generate somatic instability in non-dividing cells. Consistent with this view, MMR proteins have recently been shown to scan the genome independently of their association with replication factories and to initiate a repair reaction (51) or mediate the mutation of single bases in non-dividing cells (52). More recently, small repeat sequences extruded from the conventional B-DNA helix have been shown to activate MMR in the absence of DNA replication (53).

Our data do not preclude a role for DNA replication slippage during cell division in generating repeat size mutations, but raise a pertinent question: what is the relative contribution of replication-dependent versus cell division-independent mechanisms in generating expansions *in vivo*? Here, we have established that rates of repeat expansion in arrested cells were at least as high as those in rapidly proliferating cells, showing that DNA replication-independent pathways are capable of generating high levels of variation. It seems reasonable to assume that cell division-independent mechanisms must be operating *in vivo* to generate dramatic levels of somatic mosaicism and it is possible that DNA replication slippage during genome duplication plays only a minor role.

The data presented here definitively establish that a major mechanism exists for expanding trinucleotide repeats in mammalian cells, independent of the cell cycle. Indeed, by establishing that neither cell progression or cell cycle arrest are either necessary or sufficient to initiate instability, we further highlight the pivotal role of MMR and other DNA repair genes (e.g. *Ogg1* (17) and *Xpa* (18)) and other *cis*-acting factors in driving tissue-specific repeat instability. New insights into the expansion pathway will provide a rational basis for the development of novel therapies based on suppressing somatic expansion (2). The search for chemical modifiers of trinucleotide repeat dynamics in culture is complicated by the requirement for relatively long period of culture to reliably detect changes in the rate of repeat expansion. Long culture periods of rapidly dividing cells introduce the opportunity for stochastic variation and cell selection effects, confounding the interpretation of results. The arrested cell cultures developed here may circumvent this pitfall, reducing the selection biases. Moreover, this non-proliferative model reflects to a certain extent the cellular dynamics of the post-mitotic tissues primarily affected in the repeat expansion disorders and the primary targets for therapeutic intervention. Therefore, it provides a useful cell model to identify factors and therapeutic agents capable of modifying the dynamics of expanded CAG·CTG repeats in post-mitotic tissues.

## SUPPLEMENTARY DATA

Supplementary Data are available at NAR Online.

SUPPLEMENTARY DATA
