# Supplementary material for: Disease-associated CAG·CTG triplet repeats expand rapidly in non-dividing mouse cells, but cell cycle arrest is insufficient to drive expansion
Source: Nucleic Acids Res. 2014 Apr 21;42(11):7047–56. doi: 10.1093/nar/gku285 (PMC4066746; doi:10.1093/nar/gku285)
Supplement: SUPPLEMENTARY DATA [file supp_42_11_7047__index.html]

Disease-associated CAG·CTG triplet repeats expand rapidly in non-dividing mouse cells, but cell cycle arrest is insufficient to drive expansion — Disease-associated CAG·CTG triplet repeats expand rapidly in non-dividing mouse cells, but cell cycle arrest is insufficient to drive expansion — SUPPLEMENTARY DATA 

# Disease-associated CAG·CTG triplet repeats expand rapidly in non-dividing mouse cells, but cell cycle arrest is insufficient to drive expansion

## SUPPLEMENTARY DATA

**Files in this Data Supplement:**

- SUPPLEMENTARY DATA
