# Supplementary material for: Disease-associated CAG·CTG triplet repeats expand rapidly in non-dividing mouse cells, but cell cycle arrest is insufficient to drive expansion
Source: Nucleic Acids Res. 2014 Apr 21;42(11):7047–56. doi: 10.1093/nar/gku285 (PMC4066746; doi:10.1093/nar/gku285)
Supplement: SUPPLEMENTARY DATA [file supp_gku285_nar-00186-f-2014-File007.doc]

*SUPPLEMENTARY DATA*

Disease-associated CAG•CTG triplet repeats expand rapidly in non-dividing mouse cells, but cell cycle arrest is insufficient to drive expansion

Mário Gomes-Pereira, James D. Hilley, Fernando Morales, Berit Adam, Helen E. James, and Darren G. Monckton

**SUPPLEMENTARY FIGURES**

**
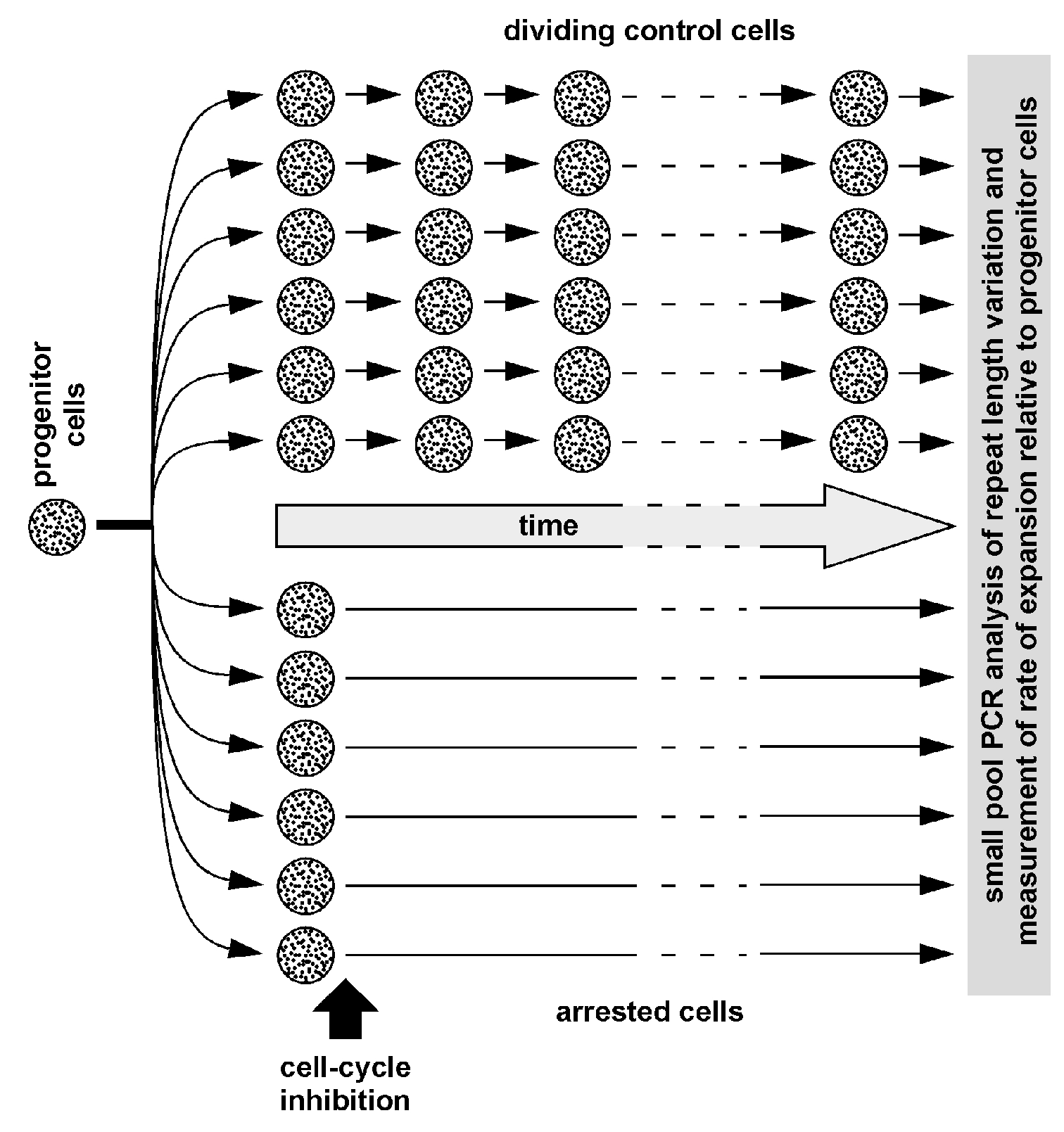
**

**Figure S1.** Experimental strategy of cell cycle arrest. The schematic illustrates the experimental strategy for each treatment with six dividing cell control cultures maintained in parallel with six arrested cell cultures all derived from a common progenitor cell culture. Cell cycle inhibition was mediated by either acute or continuous exposure to chemical inhibitors, overexpression of p16^INK4A^ or p21^WAF1^, or serum starvation.

**
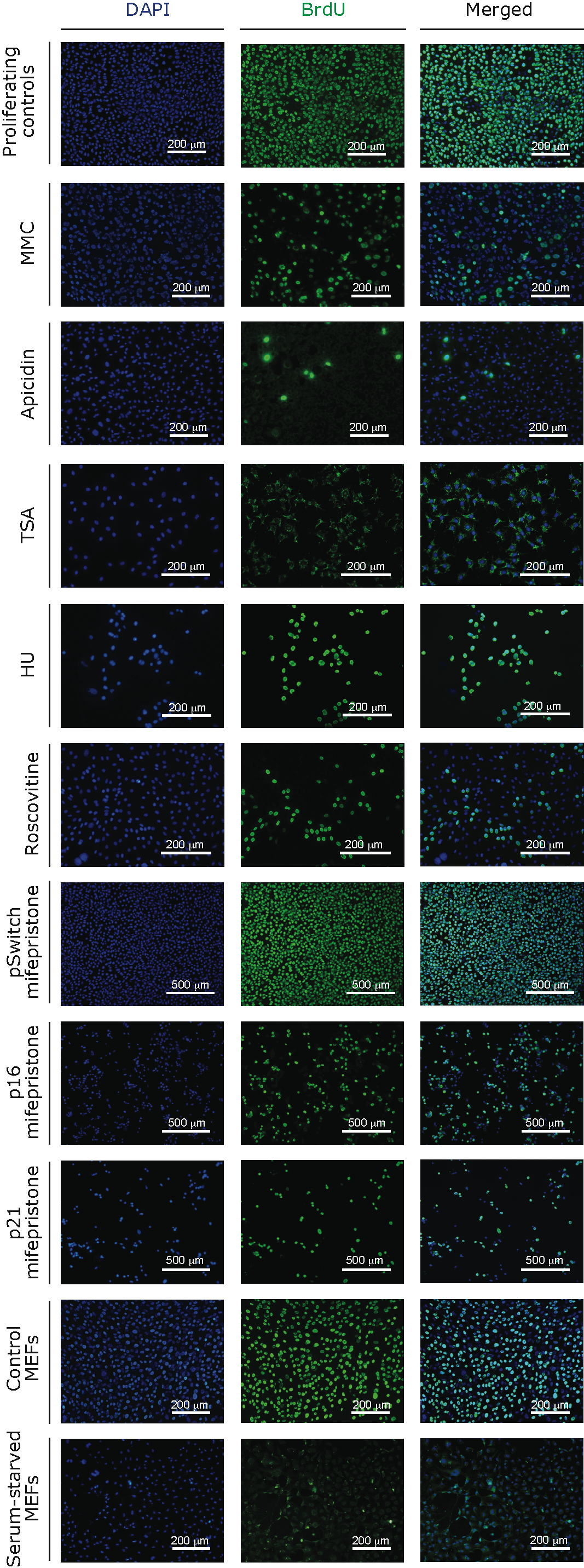
**

**Figure S2.** Immunocytochemical detection of BrdU incorporation in arrested cells. Representative low magnification images reveal the proportion of BrdU immunostaining (green) nuclei relative to total nuclei counter-stained with DAPI (blue).


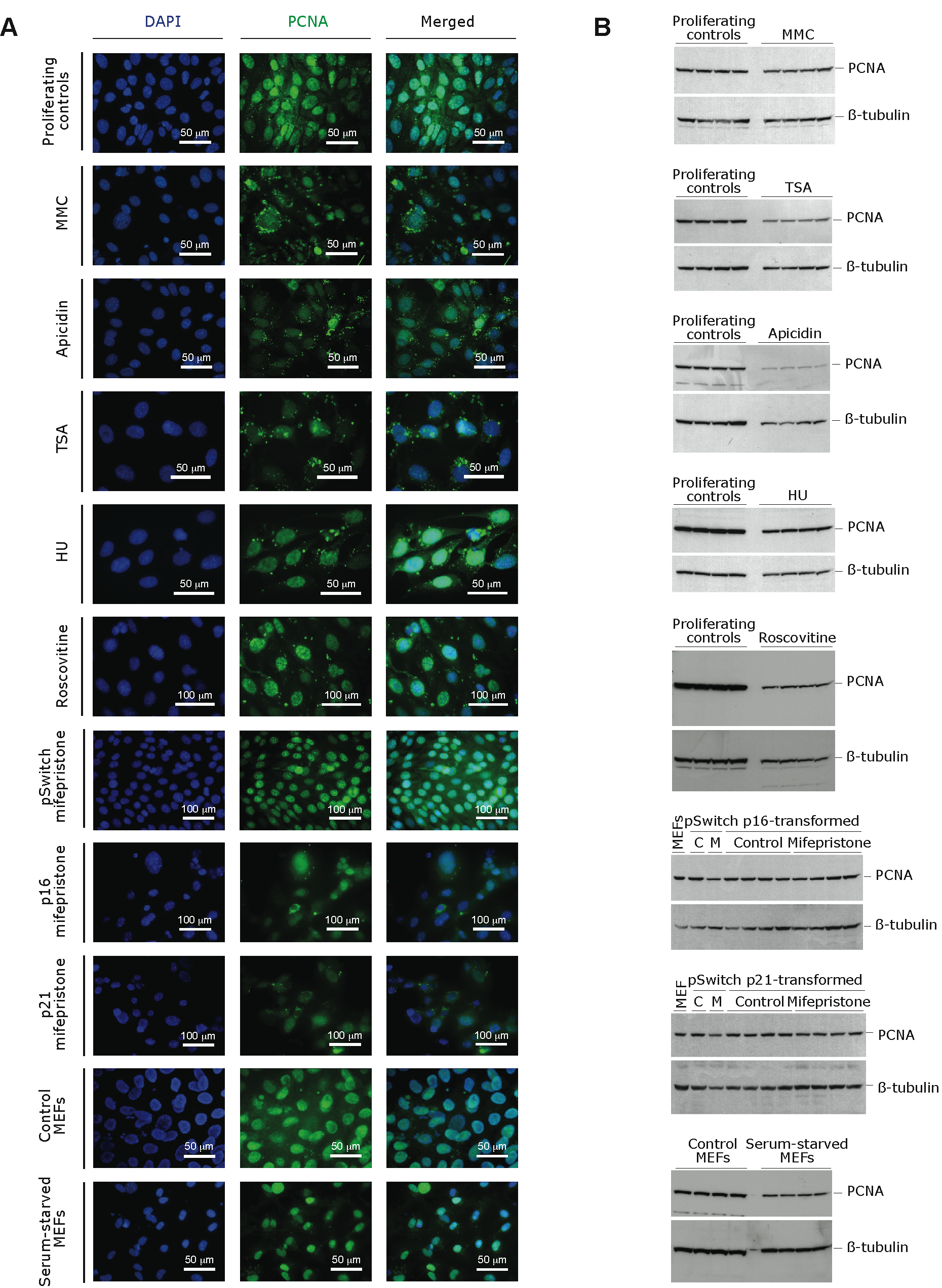


**Figure S3**. PCNA levels in arrested cells. (**A**) Low magnification images reveal the extent of PCNA immunostaining (green) in cells with DAPI counter-stained nuclei (blue). (**B**) PCNA expression in chemically-arrested cells and serum starved MEFs was quantified by western blot analysis relative to proliferating control cells. PCNA levels in cells over-expressing p16^INKA4a^ or p21^WAF1^ following mifepristone A induction were compared to PCNA expression in non-induced cells. Four protein samples collected from each culture were resolved by SDS–PAGE, electroblotted and probed with anti-PCNA and anti-β-tubulin antibodies. Protein lysates extracted from MEF cultures and pSwitch-transformed cultures, either in the absence (C) or presence (M) of mifepristone A, were included as additional controls.

**
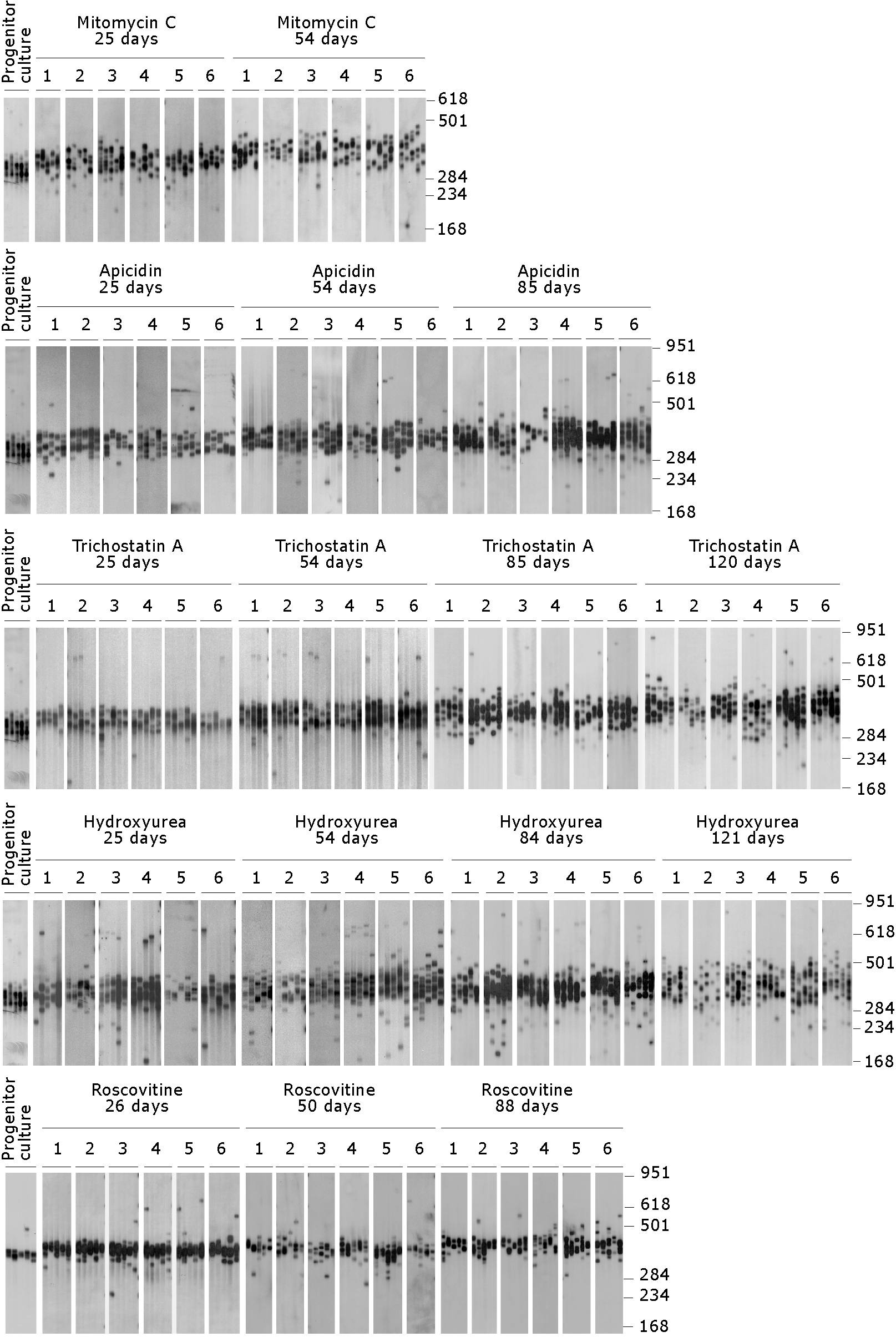
**

**Figure S4.** CAG•CTG repeat dynamics throughout cell cycle arrest. Representative SP-PCRs of the expanded CAG•CTG repeats in replicate D2763Kc2 cultures (1-6) chemically arrested for the time period indicated. Also included are the relevant progenitor culture from which all cultures were derived at day zero. The scale on the right indicates the DNA molecular weight markers converted into number of CAG•CTG repeats.


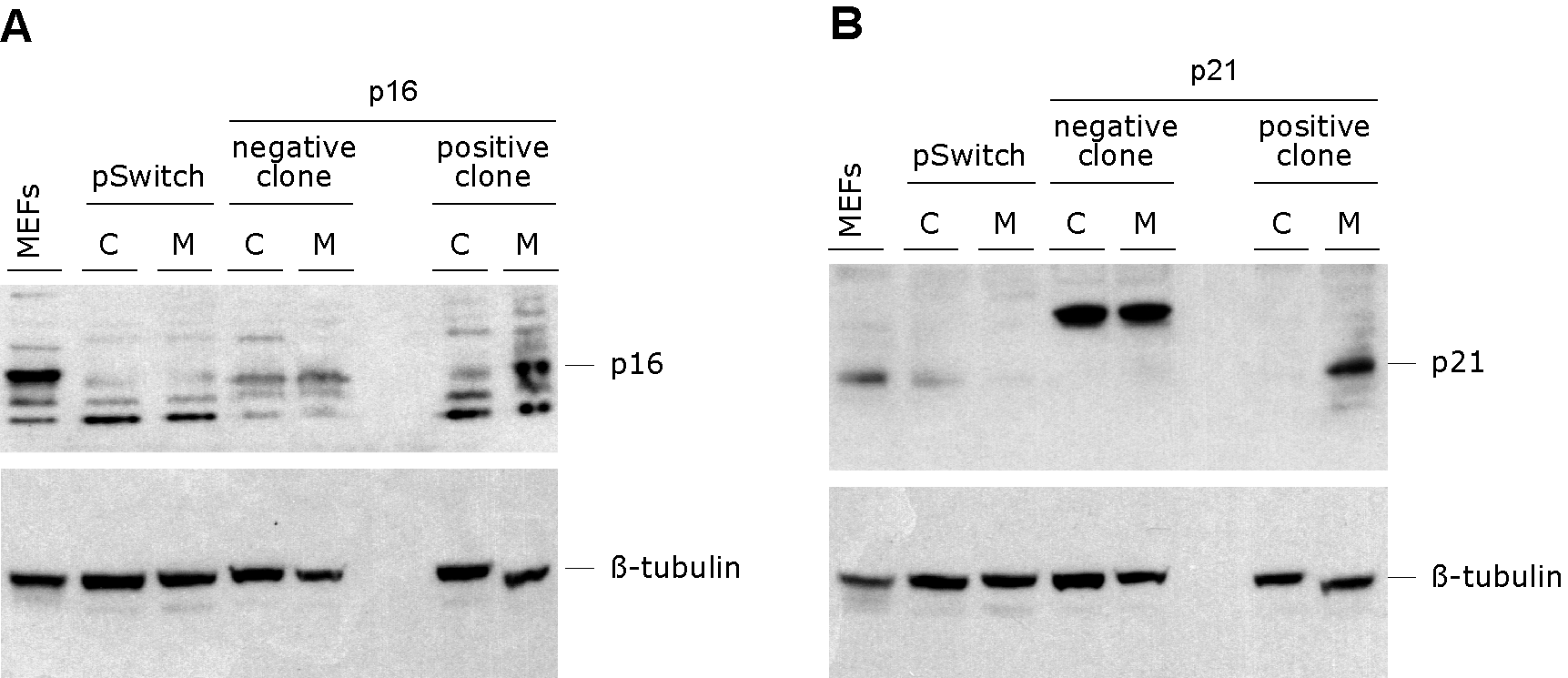


**Figure S5.** Mifepristone induced p16^INK4a^ and p21^WAF1^ expression. Western blot analysis. Protein samples were collected from single pSwitch-transformed cultures (pSwitch) or cells co-transfected with the pSwitch regulatory plasmid and pGENE/V5-His A/p16^INK4a^ (p16) (**A**), or pGENE/V5-His A/p21^WAF1^ (p21) (**B**), in the absence (C) or presence (M) of mifepristone A. The analysis of two double-transfected cell clones for each protein: a clone that does not respond to mifepristone A (negative clone), and a clone that responds to mifepristone A induction (positive clone) is shown. The positive clones overexpressing p16^INK4a^ or p21^WAF1^, were the clones selected to perform the genetic cell cycle arrest experiments. Proteins were separated on a 12% SDS–PAGE gel and electroblotted. The blots were probed with anti-p16^INK4a^, or anti-p21^WAF1^, and anti-β-tubulin antibodies. Protein extracts from primary MEF cultures were included as positive controls. Non-specific bands were detected with the antibodies directed against p16^INK4a^ or p21^WAF1^. The major bands detected in the positive controls corresponded to proteins of expected molecular weight sizes (16 and 21 kDa). The abundant polypeptide of high molecular weight detected in the p21 negative clone, may result from rearrangement events of the pGENE/V5-His A/p21^WAF1^ plasmid during cell transformation and construct integration.


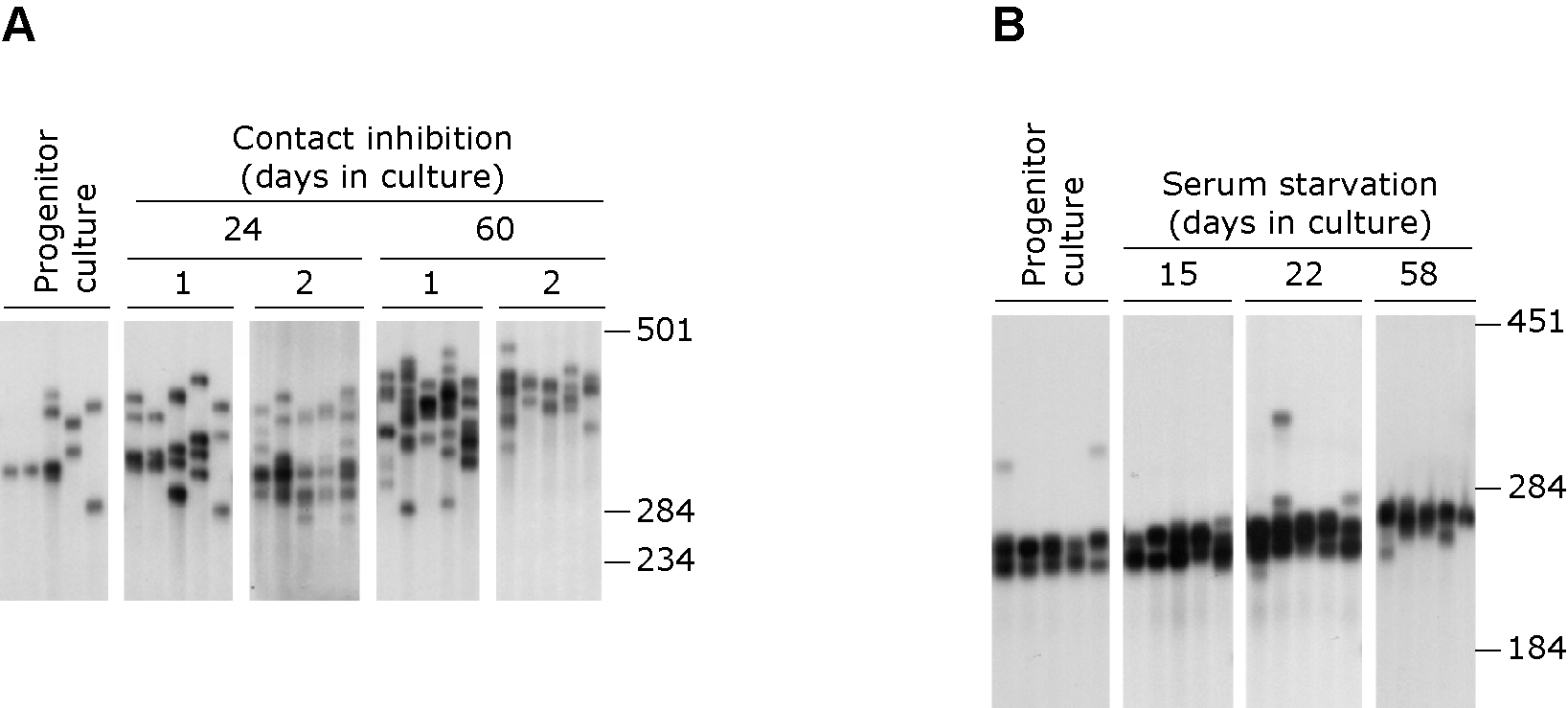


**Figure S6.** The CAG•CTG repeat continues to expand in primary *Dmt-*D kidney cells arrested under mild conditions, in the absence of chemical exposure or genetic manipulation. (**A**) SP-PCR analysis of repeat size variability in two replicate cultures, derived from the progenitor cells at day zero, and arrested by contact inhibition for the time period indicated. Marked expansion-biased instability is illustrated by the increase in the median repeat size over time. (**B**) SP-PCR analysis of the expanded repeat tract in primary kidney cultures arrested by serum starvation, for the time period indicated. The analysis shows an increment of the median repeat size over time, corroborating the continuous accumulation of repeat expansions in the absence of cell division. The scale on the right indicates the DNA molecular weight markers converted into number of CAG•CTG repeats.

**SUPPLEMENTARY METHODS**

**Plasmid construction and cell transfection.**

p16^INK4a^ and p21^WAF1^ cDNA clones were used to generate mifepristone-regulated D2763Kc2 expression clones using the GeneSwitch system (Invitrogen, cat. no. K1060-01), according to the manufacturer’s instructions. Transfections were performed with TransIT‑LT1 reagent (Mirus corporation, cat. no. 2300) and β‑galactosidase specific activity was quantified using the β‑gal assay kit (Invitrogen, cat. no. K1455‑01). Two series of double-transfected clones were screened by western blot analysis for high expression levels of p16^INK4a^ or p21^WAF1^ following a one-week induction period with 10 nM mifepristone A. The clonal cell lines showing the highest protein expression levels of p16^INK4a^ or p21^WAF1^, relative to a β‑tubulin loading control, were selected to perform this study.

**BrdU incorporation.**

Arrested cells were maintained on LabTek II 8‑well chamber slides (Nalge Nunc, cat. no. 154534) for one week and nuclear DNA synthesis and/or cell proliferation assessed by 5’-bromo-3-deoxyuridine (BrdU) incorporation and detection analysis, using the BrdU detection and labelling kit I (Roche Boehringer, cat. no. 1296736) following the manufacturer’s protocol. At least 200 individual cells were scored. Stained cells were observed using fluorescence microscopy and nuclear BrdU incorporation levels were determined as the percentage of FITC-labelled nuclei relative to DAPI-labelled nuclei.

**PCNA immunocytochemistry.**

PCNA immunocytochemistry was performed on LabTek II 8-well chamber slides (Nalge Nunc, cat. no. 154534). Cells were washed briefly with ice-cold 1X PBS, fixed with 100% (v/v) methanol at -20°C for 5 minutes and air-dried. Cells were washed three times in 1X PBS for five minutes and incubated with 10% (w/v) BSA in 1X PBS for 30 minutes at room temperature with gentle shaking, to suppress non-specific binding. Cells were incubated with 20 μg ml^-1^ rabbit FITC-conjugate anti-PCNA antibody (Santa Cruz, sc-7907 FITC) in 3% (w/v) BSA, overnight at 4°C with gentle shaking. Finally, cells were washed with four changes of 1X PBS for 5 minutes each, and examined with fluorescence microscopy.

**Equipment and settings.**

Digital RGB microscopy images were captured using a Zeiss Axiophot 2 fluorescent microscope with either 10x, 20x or 40x objective lens and a 3,090 by 3,900 pixel Axiocam CCD camera with colour image capture, with Zeiss filter set 01 [blue for fluorophores (DAPI)] and Zeiss filter set 38 [green for fluorophores (FITC)] and controlled using AxioVision (v3.0, Zeiss) software and automatic exposure settings. Scale bars were automatically added to the raw images by the AxioVision software in the red channel. X-ray film images were digitally imported into Adobe Photoshop CS (v8.0, Adobe) using a CanoScan LiDE30 flatbed scanner (Canon) and the ScanGear CS import driver set to capture a grey scale image at 300 dpi. Figures were assembled and labelled using either Canvas or FreeHand MX (v11.0, macromedia). Where images from multiple X-ray films were juxtaposed, images were aligned and scaled using the common molecular weight markers as reference points.

**Protein sample preparation and western blotting.**

Protein was extracted from cultured cells using EBC lysis buffer (50 mM Tris‑HCl, 120 mM NaCl, 0.5% (v/v) NP‑40) containing protease inhibitors (protease inhibitor cocktail for mammalian tissues, Sigma, cat. no. P8340). Aliquots of 50‑100 μg of whole cell protein lysates were resolved by electrophoresis through a NuPAGE 4‑12% Bis‑Tris Gel (Invitrogen, cat. no. NP0321) and electroblotted onto Millipore Immobilon-P membranes (Millipore, cat. no. IPVH00010) at 30 V for 2 h in an XCellII Blot Module (Novex, cat. no. EI9051) in NuPAGE Transfer Buffer (Invitrogen, cat. no. NP0006‑1). The membranes were blocked overnight at 4°C in 2.5% (w/v) or 5% (w/v) dried milk in TBST (20 mM Tris‑HCl pH 7.6, 137 mM NaCl, 0.06% (v/v) Tween-20), then incubated for two hours at room temperature in primary antibody. The membranes were washed four times for 15 min each in TBST, incubated for 1 h in secondary antibody at room temperature, and washed four times for 15 min each in TBST at room temperature. Antibody binding was visualised using SuperSignal West Pico Chemiluminescent Substrate (Pierce, cat. no. 34080). PCNA was detected using 400 ng ml^-1^ rabbit anti‑PCNA polyclonal antibody (Santa Cruz, cat. no. sc‑7907) in 5% (w/v) dried milk in TBST. p21^WAF1^ was detected using 200 ng ml^-1^ rabbit anti‑p21^WAF1^ polyclonal antibody (Santa Cruz Biotechnology, cat. no. sc‑471) in 5% (w/v) dried milk in TBST. p16^INK4a^ was detected using 400 ng ml^-1^ rabbit anti‑p16^INK4a^ polyclonal antibody (Santa Cruz Biotechnology, cat. no. sc‑1207) in 2.5% (w/v) dried milk. β‑Tubulin was detected using 200 ng ml^-1^ rabbit anti‑β‑tubulin polyclonal antibody (Santa Cruz Biotechnology, cat. no. sc‑9104). All proteins were detected using 20 ng ml^-1^ goat anti‑rabbit (Santa Cruz Biotechnology, cat. no. sc‑2004), 8 ng ml^-1^ goat anti‑mouse (Jackson ImmunoResearch , cat. no. 115-035-003) or 4 ng ml^-1^ donkey anti‑goat (Santa Cruz Biotechnology, cat. no. sc-2020) horseradish peroxidase-conjugated secondary antibodies diluted in 2.5% (w/v) or 5% (w/v) dried milk in TBST. Incubation with Restore Western Blot Stripping Buffer (Pierce, cat. no. 21059) for 20 minutes at 40°C was performed to break antibody-antigen interactions and to subsequently re-probe the same membrane. Densitometric analysis of PCNA protein expression levels was performed using Kodak Digital Science 1D software using exposures in the linear range of signal intensity.

**SP-PCR amplification.**

Each lane on the representative autoradiographs shown in the figures contains the amplification products of a single SP-PCR with ~5-20 genomic equivalents of input DNA. Each autoradiograph panel for an individual replicate culture is a cropped image derived from a separate autoradiograph and multiple images have been juxtaposed to aid comparison. The degree of repeat length variation observed in treated and control cell replicates at the time points indicated was schematically represented by box plots. The top and bottom of the boxes correspond to the third (Q3) and first quartiles (Q1), respectively and the line across the box displays the median. The lines extending from the top and the bottom of the boxes, include values that fall inside the lower and upper limits: Q1-1.5(Q3-Q1) and Q3+1.5(Q3-Q1), respectively. The median repeat length gain was determined by measuring the degree of variation in individual cultures and comparing this with the degree of variation measured in the progenitor culture. Expansion rates (in units of repeats gained per day) were calculated by dividing the median repeat gain over the number of days in culture, as previously described (28)

**Supplementary references**

28. Gomes-Pereira, M. and Monckton, D.G. (2004) Chemically induced increases and decreases in the rate of expansion of a CAG•CTG triplet repeat. *Nucleic Acids Res*, **32**, 2865-2872.
